# Social Isolation in Turkish Adolescents: Translation, Cross-Cultural Adaptation, and Validation of the Social Isolation Questionnaire

**DOI:** 10.3390/children12091122

**Published:** 2025-08-26

**Authors:** Hamide Nur Çevik Özdemir, Gülsün Ayran

**Affiliations:** 1 Department of Child Health and Disease Nursing, Faculty of Health Science, Afyonkarahisar Health Sciences University, Afyonkarahisar 03030, Türkiye; 2 Department of Child Health and Disease Nursing, Faculty of Health Science, Erzincan Binali Yıldırım University, Erzincan 24000, Türkiye; gayran@erzincan.edu.tr

**Keywords:** social isolation, adolescents, scale, mental health, cross-cultural adaptation, psychometric

## Abstract

**Objectives**: Social isolation is an important public health issue that is becoming increasingly prevalent among adolescents today. Recognizing the risk of social isolation in children and adolescents during their developmental years can contribute to the prevention of depression and anxiety disorders, as well as risky behaviors such as suicide and substance abuse. Valid and reliable measurement tools are needed to assess social isolation in adolescents. There is a lack of surveys to identify gaps in social isolation among Turkish adolescents. This study aims to adapt the Social Isolation Questionnaire (QIS) scale, which was developed for adolescents, to Turkish culture and to evaluate its psychometric properties. **Methods**: This descriptive and methodological study was conducted between July and November 2024 with 1922 adolescents. Data were collected using an introductory information form, the QIS, and the UCLA Loneliness Scale. In the evaluation of the data, explanatory and confirmatory factor analysis, Cronbach’s alpha, split-half, item–total score correlation, and test–retest analysis were used. **Results**: The average age of the adolescents was 13.97 ± 1.67 years (min = 12, max = 17). A total of 50.6% of the adolescents were male and 49.4% were female. A total of 81.2% of the adolescents had a nuclear family structure. Exploratory factor analysis revealed a three-factor structure explaining 55.97% of the variance. The factor loadings were greater than 0.30, and all fit indices were greater than 0.90. The total Cronbach’s α value of the scale was 0.83, while the values for the subdimensions ranged from 0.73 to 0.75. **Conclusions**: The Turkish version of the QIS is a valid and reliable measurement tool for assessing social isolation in adolescents. This questionnaire can be used by health professionals and researchers to identify adolescents at risk of social isolation and plan appropriate mental health interventions. This questionnaire can be used in studies focusing on adolescent mental health and well-being, contributing to the development and implementation of strategies in line with the Sustainable Development Goals.

## 1. Introduction

Adolescence is a critical stage between childhood and adulthood, characterized by rapid changes in biological, cognitive, emotional, and social areas [1]. During this period, the social environment plays a fundamental role in terms of peer relationships, the development of an individual’s identity, and psychosocial adjustment [2,3]. However, as a result of various individual and environmental factors, some adolescents withdraw from social relationships and face negative social experiences such as exclusion, loneliness, and rejection [4,5,6]. The adolescence process is considered within the framework of the concept of social isolation and is considered an important risk factor for adolescent mental health [7,8,9].

Social isolation is defined as the absence of social contact, an experience that can occur in the presence of others. In addition, social isolation is a situation where an individual stays away from social relationships, has limited social interaction, or cannot establish meaningful social connections [10,11]. The loneliness that results from this situation is a subjective reflection of perceived social isolation [10,12]. Social isolation and loneliness are closely related but distinct concepts. Loneliness is a subjective, negative emotion resulting from a perceived discrepancy between what individuals desire and what they achieve in social connectedness. Social isolation is positively correlated with loneliness. Social isolation limits an individual’s ability to interact with or within their existing social networks, which can lead to loneliness [13]. It is recommended that researchers examine loneliness through the lens of perceived social isolation to better understand how the experience of physical isolation changes during adolescence [10].

Social isolation and loneliness affect people of all ages around the world. Today, social isolation is considered an increasingly important public health problem among adolescents [14]. According to a World Health Organization report, at least one in six adolescents is socially isolated or lonely [15]. In a school-based health survey, the prevalence of social isolation among school-aged adolescents was reported as 28.2% [16]. Similarly, in a different study, the prevalence of social isolation among adolescents was reported as 10.14% [17]. In particular, the proliferation of digital technologies, social media use, global crises such as pandemics, and increasing individualization have led to an increase in the social isolation levels of adolescents [18]. This trend became even more evident during the COVID-19 pandemic. Quarantine and social distancing measures implemented during the COVID-19 pandemic have prevented adolescents from meeting their need for belonging, exacerbating feelings of loneliness and isolation. The closure of schools, sports, and social activities has limited face-to-face interaction with peers, negatively impacting adolescents’ psychological well-being and increasing the risk of depression, anxiety, and suicidal thoughts [19].

Adolescence has many specific developmental characteristics that pose special risks for perceived social isolation. These characteristics include developmental changes in peer relationships, processes of autonomy and individuation, identity exploration, cognitive maturation, social perspective acquisition, and physical maturation. These developmental factors may contribute to the emergence of psychological distress in adolescents [10,11]. Individuals differ in characteristics that lead to social isolation. Characteristics such as personality, temperament, shyness, and social reticence can lead to perceptions of social exclusion/isolation in individuals [10]. Adolescents have characteristics that put them at risk for social isolation. The process of identity search, together with autonomy expectations and individualization tendencies, leads to various changes in the social relationships of adolescents. This change may make adolescents more vulnerable to perceptions of social isolation due to their cognitive and physiological developmental characteristics [10].

Negative psychosocial experiences such as social isolation can be detrimental to children and adolescents during their developmental period [20]. Studies have emphasized that social isolation experienced during adolescence is associated with various negative psychosocial outcomes such as loneliness [21,22], depression [17,21], anxiety disorders [4], low self-esteem [7], academic failure, and risky behaviors (suicidal ideation, substance use, etc.) [5,6,22].

Considering that adolescents are a fragile population, factors that put their physical and mental health at risk need to be taken into account [23]. Therefore, determining the level of social isolation experienced by adolescents with reliable and valid tools is of great importance for both preventive mental health studies and early intervention strategies. Although social isolation is considered an important indicator of well-being and health [24], there is a lack of questionnaires in the literature designed to assess social isolation in adolescents [14].

Previous studies have often used question sets based on the researchers’ experience or surveys designed to measure other concepts indirectly related to social isolation [21,25]. This situation highlights the lack of original surveys that directly and comprehensively measure social isolation in the adolescent population, and leads to some methodological limitations in the existing literature [26].

In the literature, social isolation studies conducted with children or adolescents generally used measurement tools focusing on mental health and behaviors (UCLA Loneliness Scale, Children’s Behavior Checklist, Youth Problem Inventory, etc.) [4,27,28,29]. Additionally, Cavicchiolo et al. [30] developed the Classmates Social Isolation Questionnaire for Adolescents to measure peer relationships at school. However, these measurement tools may be insufficient to evaluate the abstract and multidimensional structure of social isolation in a comprehensive and healthy way. The number of scales that directly measure social isolation in the national literature is quite limited. In Turkey, the Social Isolation Scale was developed for adult individuals by Çelikbaş and Tatar [31]. In addition, the Social Isolation Subscale, one of the sub-dimensions of the Nottingham Health Profile adapted to Turkish by Küçükdeveci et al. [32], has also been used in various studies [33,34]. However, all of these scales were developed or adapted for the adult population and are inadequate both theoretically and psychometrically for assessing social isolation in an adolescent group. Therefore, self-report measurement tools are needed to assess social isolation in adolescents [25].

The Social Isolation Questionnaire, specifically developed by Dos Santos et al. [14] to assess social isolation in adolescents, stands out as an original measurement tool that fills one of the important gaps in the literature with its multidimensional structure and psychometric validity and evidence of reliability. This questionnaire allows for a comprehensive assessment of adolescents’ social ties, family relationships, and environmental interaction levels [14].

Social isolation experienced during adolescence can cause mental health problems in the long term and negatively affect an individual’s participation in society. Therefore, accurate and reliable assessment of social isolation in adolescents is of critical importance not only for individual health but also for the sustainability of public health [15,22]. Health professionals and nurses, in particular, play key roles in protecting and strengthening the mental health of adolescents; in this regard, they need valid and reliable measurement tools to determine the risk of social isolation. Nurses can use tools such as the Social Isolation Questionnaire to detect risk for social isolation in adolescents. In Türkiye, which has a significant adolescent population, there is a continuing need for standardized social isolation questionnaires specifically designed for adolescents and with proven validity and reliability. Within our literature review, no measurement tool specifically developed and culturally adapted to assess social isolation among adolescents in Turkey was found. Strengthening adolescent mental health is one of the critical areas addressed within the Sustainable Development Goals [35].

In this context, the aim of this study is to translate the Social Isolation Questionnaire for Adolescents into Turkish, to adapt it to Turkish culture, and to evaluate its psychometric properties. In this regard, the adapted survey aims to contribute to the early identification of at-risk adolescents and the establishment of preventive and intervention-oriented mental health practices.

## 2. Materials and Methods

### 2.1. Study Design

This study used a methodological, descriptive, and correlational design. Adaptation of the survey to Turkish culture was carried out within the scope of good practice guidelines used for scale adaptation [36,37]. The study was reported using the “Guidelines for Reporting Reliability and Agreement Studies (GRRAS)” [38].

### 2.2. Study Setting and Participants

This study was conducted between July and November 2024 in the city center of Erzincan province in the Eastern Anatolia Region of Turkey. The target population of the study consisted of 7th-, 8th-, 9th-, 10th-, 11th-, and 12th-grade adolescents studying in second-stage (secondary school) and third-stage (high school) schools affiliated with the National Education Directorate in the city center of Erzincan. A total of six schools, three middle schools and three high schools, with similar socioeconomic and cultural characteristics were included in the study. (These schools were affiliated with the state and did not include private schools.) Participants were selected from these six schools using a stratified sampling method, taking into account school type and grade level variables. The socioeconomic level of the participants is close to the national average according to the data of the Turkish Statistical Institute (TUIK) and represents the middle-income group.

The sample size was determined by taking into account the criteria suggested for factor analysis in the literature. In the literature, when the sample size recommended for factor analysis is categorized, samples of 100 participants are considered as inadequate, up to 200 as moderately adequate, up to 300 as good, up to 500 as very good, and up to 1000 as excellent. For this reason, experts recommend that researchers work with samples of 500 or more participants if possible [39,40]. Another suggested method is the three rules called the 5 s, 10 s and 100 s rules [41,42]. Since the QIS scale consists of 17 items, the number of participants per item was calculated as 100 and the study was planned to include at least 1700 adolescents.

After obtaining the necessary permissions from school administrators, students in designated classes from schools were informed face-to-face about the purpose and process of the study. The aim was to reach 2000 adolescents (participants) studying in these schools. No advertising or financial incentives were given to participants. First, a pilot study was conducted with 30 adolescents to evaluate the readability and clarity of the questionnaire form, and these data were not included in the main sample. The surveys were administered face-to-face by researchers using the paper-and-pencil technique. A total of 48 surveys with low reliability, incomplete data, and most of the questions unanswered were not included in the study. As a result, survey data of 1922 adolescents were evaluated. The overall response rate was calculated as 98.5% using the formula (1.970/2.000) × 100.

The ages of the adolescents included in the study ranged from 12 to 17, with a mean age of 13.97 ± (1.67) years. Of the participants, 25.2% were 12 years old (7th grade), 17.4% were 13 years old (8th grade), 20.7% were 14 years old (8th grade), 11.6% were 15 years old (10th grade), 14.8% were 16 years old (11th grade), and 10.4% were 17 years old (11th grade).

Exclusion criteria included adolescents who refused to participate in the study, those for whom parental consent could not be obtained, and those with any intellectual disability or psychiatric disorder registered with the school guidance service and confirmed by parental reports.

### 2.3. Data Collection Tools

Data were collected with a descriptive information form, the QIS in adolescents, and the UCLA Loneliness Scale.

#### 2.3.1. Information Form

This form included demographic questions such as age, gender, family type, place of residence, and number of siblings.

#### 2.3.2. Social Isolation Questionnaire (QIS)

This questionnaire was developed by Dos Santos et al. [14] to assess social isolation in adolescents. The questionnaire consists of 17 items and three sub-dimensions. The dimensions were feeling of loneliness, friendship, and family support. The questionnaire was constructed using Likert scales scored between 0 and 3. The first category is low social isolation score (0) and the fourth category (3) is high social isolation score. In the original questionnaire, the overall internal consistency as measured by Cronbach’s alpha (α) was 0.85, while the Cronbach’s alpha (α) values for the subscales of feeling of loneliness, friendship, and family support were 0.81, 0.76, and 0.68, respectively (Supplementary Materials).

#### 2.3.3. UCLA Loneliness Scale-Short Form (ULS-8)

This scale was developed by Hays and DiMatteo [43] to determine the loneliness levels of adolescents. The UCLA Loneliness Scale-short form (ULS-8) items reflect perceived social isolation as a representation of individual loneliness. The scale is used to assess feelings of loneliness in individuals from adolescence to adulthood. Turkish adaptation of the scale was conducted by Yıldız and Duy [44]. The Turkish form of the scale consists of a single dimension and 7 items. The responses are in four-point Likert-type scales: ‘(1) Never, (2) Rarely, (3) Sometimes and (4) Often’. The Cronbach’s alpha coefficient of the scale is 0.74. In adolescents, low scores correspond to low feelings of loneliness and high scores correspond to more severe feelings of loneliness.

### 2.4. Procedures

In testing the validity and reliability of the QIS questionnaire, the following application steps were performed.

#### 2.4.1. Translation

Permission was obtained from the owner of the scale via e-mail before starting the study. The scale was translated from English to Turkish by two linguists. The researchers reviewed the translations. It was then translated back from Turkish to English by a linguist who had not yet seen the scale. The Turkish form was evaluated semantically, structurally, and conceptually by the researchers and the Turkish language expert, and the scale was finalized after the necessary corrections were made [45].

#### 2.4.2. Specialist Opinions

It is recommended to obtain the opinions of at least three experts for the content validity of the questionnaire [46]. In this study, the opinions of five experts (clinical expert psychologist, guidance and psychological counselor, school nurse, and two child nursing academicians working in this field) were obtained for the Turkish questionnaire form. The original questionnaire and translated versions were sent to the experts via e-mail. Experts were informed about the relevant measures and concepts. Content validity of the scale was calculated using the Polit and Beck Content Validity Index (CVI) [47].

#### 2.4.3. Pilot Test

After receiving expert opinions, the questionnaire was piloted on 30 adolescents with similar characteristics to the sample [48]. The comprehensibility of the questionnaire was found sufficient in the pilot test. The adolescents in the pilot test were not included in the sample.

### 2.5. Data Collection Process

After obtaining ethics committee and institutional approvals, data were collected face-to-face using a pen-and-paper method in a classroom setting. Researchers visited the institutions on days and times specified by school administrators and collected data. Data were collected in a way that did not disrupt students’ academic and class schedules. Surveys were distributed to participants, who were asked to complete them independently. Data collection time averaged 5–10 min. No personal data was collected during the data collection process.

### 2.6. Statistical Analysis

IBM SPSS Statistics for Windows (Version 25.0. Armonk, NY, USA: IBM Corp) and IBM AMOS 21 package programs were used for data analysis. Frequency, percentage, mean, and standard deviation statistics were used to analyze descriptive data. CVI was used for the content validity of the questionnaire. The construct validity of the questionnaire was evaluated by explanatory and confirmatory factor analysis (CFA).

The reliability of the questionnaire was evaluated with the Cronbach’s alpha reliability coefficient and halving method. For item discrimination, a 27% lower and upper item analysis was performed. AVE (average variance extracted) and CR (composite reliability) values were also calculated. Correlation analysis was used for criterion-related validity and test–retest reliability. *p* < 0.05 was considered statistically significant.

### 2.7. Ethical Considerations

Permission was obtained from the owner of the scale [14] via e-mail for the Turkish validity and reliability study of the scale. Approval was obtained from the Non-Intervention Scientific Research Ethics Committee of the university (Protocol No: 2024/5). Institutional permission was obtained from the Directorate of National Education for the implementation of the study in schools. The adolescents participating in the study were informed about the study. Verbal and written consent of the adolescents was obtained. School administration and parents of the adolescents were informed and their consent was obtained. The study was conducted in accordance with the ethical rules specified in the Declaration of Helsinki.

## 3. Results

Within the scope of the study, the data set was divided into two groups and exploratory factor analysis (EFA) and confirmatory factor analysis (CFA) were conducted in separate groups. The mean age of the participants was 13.97 (±1.67) years (min = 12, max = 17). A total of 50.6% of the adolescents were male and 49.4% were female. The demographic characteristics of the participants in the EFA and CFA groups, such as age distribution, gender, family type, place of residence, and presence of siblings, were compared. There was no significant difference between the groups in terms of demographic variables (*p* > 0.05). The demographic characteristics of the adolescents are presented in detail in Table 1. 

According to the data in Table 2, the mean scores of the scale items vary between 0.66 and 2.32. The item–total score correlations of the scale are between 0.37 and 0.62, and it is seen that all items are compatible with the whole scale. However, the correlation value of item 2 (−0.08) is negative and low. This finding suggests that item 2 should be removed.

According to Table 3, the KMO test value is 0.860 and the sample size is sufficient for factor analysis. According to Bartlett’s Test of Sphericity (χ2 = 3581.914; *p* = 0.000), the chi-square value obtained was significant and suitable for factor analysis.

The EFA results using principal components analysis revealed three subscales of the QIS. The first scale of the scale was named “friendship”, the second scale was named “family support”, and the third scale was named “feeling of loneliness”. Eigenvalues of the factors were calculated as 4.467, 1.627, and 1.182, respectively. The factor loadings of 13 items in the scale were above 0.40. The 1st and 11th items in the scale were removed from the scale as they were overlapping items, and the 19th item was removed from the scale as it did not load under any scale. The total variance explained was 55.97% (Table 3).

**Figure 1:**
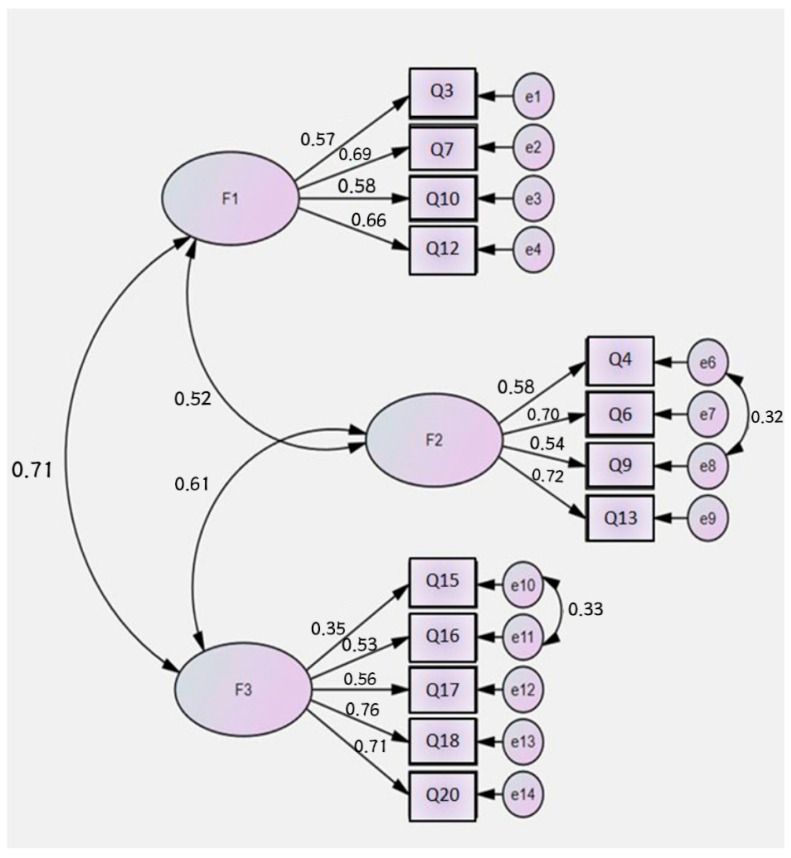
The confirmatory factor analysis results of the QIS.

According to the results of the confirmatory factor analysis of the Turkish version of the QIS questionnaire, the goodness-of-fit indices of the model showed excellent fit with a χ2 (Cmin/df) of 3.149 and a root mean square error of approximation (RMSEA) of 0.047 [49,50]. The model fit indices were SRMR = 0.037, CFI = 0.961, GFI = 0.970, AGFI = 0.955, IFI = 0.961, TLI = 0.949, and NFI = 0.944. These values showed an excellent fit of the three-factor structure of the QIS (Table 4). The factor loadings of the QIS questionnaire ranged from 0.35 to 0.76 (Figure 1).

### 3.1. Reliability

The Cronbach’s alpha values of the subscales of the QIS questionnaire were 0.73 for loneliness, 0.75 for friendship, and 0.74 for family support. The total Cronbach’s alpha value of the whole scale was found to be 0.83. Detailed findings obtained from the halving analysis are presented in Table 5.

### 3.2. Test–Retest Analysis

Test–retest analyses of the QIS were performed with 50 adolescents at 4-week intervals, and it was determined that there was no statistically significant difference between the mean scores of the two measurements (*p* > 0.05).

When Table 6 was analyzed, it was found that there was a statistically significant and strong positive relationship between the QIS questionnaire and the ULS-8 (r = 0.540, *p* < 0.01). In addition, a very high (r = 0.932, *p* < 0.01), high (r = 0.745, *p* < 0.01), and moderate (r = 0.664, *p* < 0.01) positive relationship was found between the QIS questionnaire and the feeling of loneliness subscale, friendship subscale, and family support subscale, respectively. Significant correlations were also found with the ULS-8, supporting that the measured construct is consistently related to the expected psychological variables.

## 4. Discussion

In this study, the self-report-based QIS questionnaire designed to assess social isolation in adolescents was adapted to the Turkish population and its psychometric properties were evaluated. Construct and content validity methods were used for validity analyses. For content validity, the suitability of the Turkish version of the questionnaire for the target language and culture was evaluated by experts in the field. For agreement between expert opinions, I-CVI and S-CVI values are expected to be above 0.80 [47]. In this study, both I-CVI and S-CVI values were above 0.80. These results revealed agreement between the experts, the adequacy of the scale, and its content validity.

Bartlett’s test of sphericity and KMO were used to analyze the adequacy of the sample and the suitability of the data for factor analysis. In the literature, it is stated that Bartlett’s test of sphericity should be statistically significant and the KMO value should be at least 0.60 before factor analysis can be performed [36,51]. In this study, the Bartlett’s test result was significant and the KMO value was >0.80, indicating that the data were suitable and sufficient for factor analysis. In this regard, the sample size and data set of this study were similar to the original scale developed by Dos Santos et al. [14].

Construct validity is a type of validity that explains whether the structure of the scale is consistent with the theoretical concept and structure. Factor analysis is a frequently used method to assess construct validity [52]. In exploratory factor analysis, eigenvalues of one and above are accepted to determine the number of factors [53]. Accordingly, it was found that the questionnaire consisted of three sub-dimensions. The three-factor scale explained 55.97% of the total variance. In the literature, it is stated that the variance explained in multidimensional scales should be between 40.0% and 60.0%. The higher the total variance, the stronger the construct validity of the scale [53]. The values obtained in this study were similar to the values in the original study [14]. These results support the construct validity of the QIS.

According to the results of the EFA, the factor loadings of the three subscales ranged between 0.55 and 0.79. The loading value obtained in factor analysis is the critical value that determines whether an item belongs to a particular sub-factor. An item with a factor loading < 0.30 should be removed from the questionnaire [54]. In this study, one item with a factor loading < 0.30, two overlapping items with high loadings on more than one factor, and one item that could not load under any dimension were removed. Finally, according to the final exploratory factor analysis results, the scale included 13 items grouped under three factors. The EFA factor values in this study were similar to those in the original study [14]. The findings showed that the three-dimensional structure of the scale was appropriate for the Turkish sample.

CFA confirmed the three-factor structure identified by EFA in different sample groups. CFA showed that the factor values for the three subscales of the QIS ranged from 0.35 to 0.76. According to the literature, CFA fit indices should be above 0.90, RMSEA should be below 0.08, and χ2/df should be less than five [41]. For all subscales of the QIS, factor values were >0.30, fit indices were >0.90, and RMSEA was <0.08. The CFA results in this study are consistent with the criteria stated in the literature. Unfortunately, we could not compare our results because the original study [14] did not include CFA results. The EFA and CFA results in this study reveal that the construct validity of the scale was sufficient and that it is a valid instrument that can be used for the Turkish population.

In this study, the Cronbach’s α coefficient of the scale was 0.83 and the Cronbach’s α coefficients of all sub-dimensions were > 0.70. These results show that the scale items have a homogeneous structure and the scale has good reliability [55]. In the study of Dos Santo et al. [14], the total Cronbach’s α value of the original scale was found to be >0.80. In this context, the scale in this study is similar to the original structure and has strong internal consistency.

In scale adaptation studies, the halving method is frequently used to test internal consistency. In the split-half test reliability analysis of the scale in our study, the correlation coefficient between the two halves was >0.70, and the Spearman–Brown and Guttman Split-Half coefficients were >0.80 [51,55]. The results obtained from the reliability analyses revealed that the scale was reliable.

Response bias is one of the factors affecting the reliability of scales. In this study, Hotelling’s T^2^ test was used to determine the response bias of adolescents. According to the test results, there was no response bias and the responses of the adolescents were homogenous. Therefore, the results showed that the scale was reliable [40]. Since there was no evaluation of response bias in the original study [14], no comparison could be made.

In the study, the item–total score analysis method was used to explain the relationship between the scores of the items and the total score of the scale [53]. The minimum value should be at least 0.30 and positive [56]. In this study, item–total test correlation values for QIS varied between 0.419 and 0.588. According to this result, all items in the scale are correlated with each other. In order to evaluate the discrimination of the scale, the mean scores of the lower 27% and upper 27% groups were compared by independent samples t-test. As a result of the analysis, a significant difference was found between the groups, which showed that the discriminative power of the scale was high. Unfortunately, we could not compare our results because data on the item–total score correlation of the factors in the original scale study were not provided. The results of the reliability analysis revealed that the scale had internal consistency and was reliable.

One of the most valid ways to prove the invariance of a scale is the test–retest method. No statistically significant difference between applications of the test and a high level of correlation indicate that invariance is achieved [40,57]. In this study, a subgroup of 50 adolescents completed the scale at 3-week intervals to assess the test–retest reliability of the scale. The results revealed that there was no difference between the two measured times and the scale showed consistency and stability over time [40,57]. Similarly, there was no systematic difference between the measurements in the original study [14].

Criterion-related validity is assessed when the adapted scale shows significant relationships with another scale measuring similar constructs. In this context, a statistically significant and positive correlation was found between the QIS scale used in the study and the UCLA Loneliness Scale (r = 0.540). This finding supports the criterion-related validity of the scale. Similarly, in the original scale study, there was a similar level of correlation (r = 0.543) between the QIS and the CES-D questionnaire. The results obtained indicate that the QIS scale is a valid instrument.

The QIS scale offers several advantages as the first measurement tool adapted to Turkish culture for adolescents, having been tested for validity and reliability. Its linguistically and culturally appropriate adaptation allows for a more accurate and meaningful assessment of social isolation in adolescents. This adaptation will not only facilitate reliable data collection in future studies but may also facilitate comparisons across different cultural contexts. It is also believed that it may contribute to the development of evidence-based interventions aimed at reducing social isolation and promoting psychosocial well-being in adolescents.

## 5. Limitations

The fact that this study was conducted through a careful and systematic translation and cultural adaptation process demonstrates methodological rigor. Additionally, psychometric evaluations, including both exploratory and confirmatory factor analyses, were conducted in the study.

The survey is the first culturally adapted measurement tool to assess social isolation in adolescents aged 12–17. Despite these promising results, there are some limitations that should be considered. The QIS is a self-report questionnaire; therefore, adolescents may not have made entirely objective assessments or fully reflected their true feelings when completing it. The retrospective nature of the survey questions may pose a risk of recall bias. Furthermore, given the sensitivity of issues surrounding social isolation, the risk of social desirability should also be considered. Furthermore, the study was conducted in a single province, which may limit the generalizability of the findings to adolescents in other regions or cultural contexts.

## 6. Conclusions

This study revealed that the Turkish adaptation of the QIS is a valid and reliable measurement tool for assessing social isolation in adolescents. The measurement tool can be safely used by health professionals, nurses, and researchers to determine the risk of social isolation in adolescents. The findings obtained may form the basis for evaluating adolescents’ perceptions of social isolation and for developing preventive and remedial intervention programs to reduce this risk. In the future, experimental and applied studies aimed at reducing social isolation should be planned; the effectiveness of such interventions can be evaluated through the aforementioned survey.

The Turkish version of the QIS contributes to filling the gap in measurement tools in this area and provides new information to understand social isolation in adolescents. The data obtained using this survey will guide the development of public health policies and the structuring of psychosocial support programs for adolescents.

In future studies, it is recommended that this questionnaire be applied to adolescent subgroups with different age groups, socioeconomic levels, and social risk factors, and validity and reliability analyses be conducted. In the context of Türkiye, regional differences in social isolation should be examined by applying the QIS scale in different socioeconomic regions and rural–urban settlements. Additionally, applying the scale to special at-risk groups (e.g., immigrant or disadvantaged youth) may contribute to the development of policy and intervention programs. At the international level, adapting the QIS scale to other cultures and conducting comparative studies in different countries can reveal cross-cultural similarities and differences in social isolation in adolescents. Additionally, we recommend investigation of the long-term effects of social isolation on adolescents’ mental health, academic success, and social development through longitudinal studies.

## Figures and Tables

**Table 1:** Demographic characteristics of the participants (Group EFA and CFA comparison).

		EFA	CFA	Statistical Test
Characteristics		$\bar{x}$ ± SD	$\bar{x}$ ± SD	
Age		14.04 ± 1.68	13.91 ± 1.67	
		n (%)	n (%)	
Sex	Female	468 (48.7)	482 (50.2)	X^2^ = 0.408; P = 0.523
	Male	493 (51.3)	479 (49.8)	
Family type	Nuclear family	780 (81.2)	777 (80.9)	X^2^ = 3.122; P = 0.210
	Extended family	126 (13.1)	143 (14.9)	
	Broken family	55 (5.7)	41 (4.3)	
Type of Residence	City center	743 (77.3)	765 (79.6)	X^2^ = 1.825; P = 0.401
	District	134 (13.9)	115 (12.0)	
	Village	84 (8.7)	81 (8.4)	
Presence of Siblings	Yes	890 (92.6)	880 (91.6)	X^2^ = 0.714; P = 0.398
	No	71 (7.4)	81 (8.4)	

$\bar{x}$, *mean*; **χ**^2^, chi-square.

**Table 2:** Descriptive statistics and item total score correlations of the items of the QIS.

Items	$\bar{x}$ ± SD	Corrected Item–Total Correlation	Cronbach’s Alpha If Item Deleted
1	1.35 ± 1.10	0.473	0.809
2	2.32 ± 1.60	−0.089	0.863
3	0.78 ± 0.76	0.513	0.809
4	1.17 ± 0.94	0.467	0.810
5	1.11 ± 0.97	0.480	0.809
6	0.94 ± 0.92	0.460	0.810
7	1.49 ± 0.99	0.417	0.813
8	0.93 ± 0.86	0.412	0.813
9	1.36 ± 0.81	0.510	0.809
10	0.71 ± 0.85	0.479	0.810
11	0.89 ± 0.90	0.514	0.808
12	1.23 ± 1.04	0.370	0.816
13	0.84 ± 0.89	0.457	0.811
14	0.75 ± 0.89	0.488	0.809
15	0.84 ± 0.85	0.624	0.802
16	0.70 ± 0.93	0.530	0.806
17	0.66 ± 0.89	0.568	0.805

**Table 3:** Results of exploratory factor analysis.

Factors	Items	Factor Loading	λ	Variance
Friendship	3	0.662	4.467	19.33%
	6	0.759		
	8	0.695		
	10	0.740		
Family Support	4	0.776	1.627	18.64%
	5	0.779		
	7	0.739		
	11	0.650		
Loneliness	12	0.725	1.182	17.99%
	13	0.791		
	14	0.557		
	15	0.562		
	17	0.621		
Total explained variance		55.97%		
Kaiser–Meyer–Olkin coefficient		0.860		
Bartlett’s test of sphericity		0.000		

λ: *eigenvalue*; Χ^2^(78) = 3581.914.

**Table 4:** Model fit indices of the QIS.

Scales	χ2/df	RMSEA	SRMR	CFI	GFI	AGFI	IFI	TLI	NFI
QIS	3.149	0.047	0.037	0.961	0.970	0.955	0.961	0.949	0.944

RMSEA, root mean square error of approximation; SRMR, standardized root mean square residual; CFI, comparative fit index; GFI, goodness of fit index; AGFI, adjusted goodness of fit index; IFI, incremental fit index; TLI, Tucker –Lewis index; NFI, normed fit index; **χ**^2^, chi-square; DF, degree of freedom; QIS, Social Isolation Questionnaire.

**Table 5:** Results of the reliability analysis of the QIS and subscale (n = 900).

	Total Scale Score	Loneliness Subscale	Friendship Subscale	Family Support Subscale
Cronbach’s α	0.83	0.73	0.75	0.74
Cronbach’s α of the first half	0.68			
Cronbach’s α of the second half	0.69			
Spearman–Brown	0.87			
Guttman Split-Half	0.87			
Correlation between two halves	0.78			

**Table 6:** Relationship between QIS and ULS-8.

		Loneliness	Friendship	Family Support	QIS	ULS-8
Loneliness	r	1				
	*p*					
Friendship	r	0.543 **	1			
	*p*	0.000				
Family support	r	0.442 **	0.405 **	1		
	*p*	0.000	0.000			
QIS	r	0.932 **	0.745 **	0.664 **	1	
	*p*	0.000	0.000	0.000		
ULS-8	r	0.537 **	0.396 **	0.278 **	0.540 **	1
	*p*	0.000	0.000	0.000	0.000	

** *p* < 0.01.

## Data Availability

The original contributions presented in this study are included in the article. Raw data supporting the conclusions of this article will be made available by the authors on request.

## Supplementary Materials

The following supporting information can be downloaded at: https://www.mdpi.com/article/10.3390/children12091122/s1, Supplementary_Original scale form, Supplementary_Turkish scale form.

## Abbreviations

The following abbreviations are used in this manuscript:

AGFI	Adjusted Goodness of Fit Index
ANOVA	One-way Analysis of Variance
CFA	Confirmatory Factor Analysis
CFI	Comparative of Fit Index
CVI	Content validity index
CVR	Content Validity Ratios
DF	Degree of Freedom
EFA	Exploratory Factor Analysis
GFI	Goodness of Fit Index
IFI	Incremental Fit Index
KMO	Kaiser–Meyer–Olkin
NFI	Normed Fit Index
QIS	Social Isolation Questionnaire
RMSEA	Root Mean Square Error of Approximation
SD	Standard Deviation
SPSS	Statistical Package for the Social Sciences
SRMR	Standardized Root Mean Square Residual
TLI	Tucker–Lewis Index
ULS-8	University of California Loneliness Scale-Short Form
WHO	World Health Organization
