# Supplementary material for: Social Isolation in Turkish Adolescents: Translation, Cross-Cultural Adaptation, and Validation of the Social Isolation Questionnaire
Source: Children (Basel). 2025 Aug 26;12(9):1122. doi: 10.3390/children12091122 (PMC12469003; doi:10.3390/children12091122)
Supplement: Supplementary file 1 [file children-12-01122-s001.zip › Supplementary_Original scale form.pdf]

Supplementary –Social Isolation Questionnaire (Original form)

Reference: Dos Santos SJ, Soares FC, Gaoua N, Rangel Junior JF, Lima RA, de Barros MVG. Development and validation of a scale to measure social isolation in adolescents. J Res Adolesc. 2024;34(3):1069-77. https://doi.org/10.1111/jora.12952

1. How often have you felt lonely during the last 12 months?	( ) never	( ) rarely	( ) sometimes	( ) most of the time	( ) always
2. How many close friends do you have? (close friends are people you can count on to listen or help you if you need it)	( ) none	( ) 1	( ) 2	( ) 3	( ) 4 or more
3. To what extent do you consider the number of friends you have sufficient and who can you count on to listen or help you if you need it?	( ) completely insufficient	( ) insufficient	( ) sufficient	( ) completely sufficient	
4. To what extent do you consider the number of relatives you have sufficient as those who you can count on to listen to you or help you if you need it?	( ) completely insufficient	( ) insufficient	( ) sufficient	( ) completely sufficient	
5. To what extent do you consider your relationships and social contacts to be superficial?	( ) completely superficial	( ) superficial	( ) a little superficial	( ) not at all superficial	
6. To what extent do you consider adequate the frequency with which you can talk to your parents about your problems and feelings?	( ) completely inadequate	( ) inadequate	( ) adequate	( ) completely adequate	
7. To what extent do you consider adequate the frequency with which you can talk with your closest friends about your problems and feelings?	( ) completely inadequate	( ) inadequate	( ) adequate	( ) completely adequate	
8. To what extent do you consider adequate the frequency with which you can talk to teachers and school staff about your problems and feelings?	( ) completely inadequate	( ) inadequate	( ) adequate	( ) completely adequate	
9. To what extent do you consider satisfactory the frequency with which your relatives consult you or seek you out to talk when they need to make an important decision or when they have problems?	( ) completely inadequate	( ) unsatisfactory	( ) satisfactory	( ) completely satisfactory	
10. To what extent do you consider the frequency with which your friends consult you or seek you out to talk to you when they need to make an important decision or when they have problems?	( ) completely unsatisfactory	( ) unsatisfactory	( ) satisfactory	( ) completely satisfactory	
11. To what extent do you consider that your opinions and ideas are not shared or are of no interest to the people around you?	( ) no one is interested and/or shares my opinions and ideas	( ) a few people are interested and/or share my opinions and ideas	( ) most people are interested and/or share my opinions and ideas	( ) everyone is interested and/or share my opinions and ideas	
12. To what extent do you consider the quality of the relationships you have with your closest friends to be satisfactory?	( ) completely unsatisfactory	( ) unsatisfactory	( ) satisfactory	( ) completely satisfactory	
13. To what extent do you consider the quality of the relationships you have with your family members to be satisfactory?	( ) completely unsatisfactory	( ) unsatisfactory	( ) satisfactory	( ) completely satisfactory	
14. To what extent do you consider the quality of the relationships you have with your teachers and other school employees to be satisfactory?	( ) completely unsatisfactory	( ) unsatisfactory	( ) satisfactory	( ) completely satisfactory	

15. How unhappy do you feel about doing things alone or feeling excluded, isolated or blocked by others?	( ) completely unhappy	( ) unhappy	( ) a little unhappy	( ) I don't feel unhappy
16. To what extent do you consider yourself excluded, isolated or blocked by others?	( ) completely excluded, isolated, or blocked	( ) I feel very excluded, isolated, or blocked	( ) I feel a little excluded, isolated, or blocked	( ) I don't feel excluded, isolated, or blocked
17. To what extent does your ability to approach and communicate with the people around you bother you?	( ) it bothers me very much	( ) it bothers me	( ) it bothers me a little	( ) It doesn't bother me at all
18. Overall, to what extent do you consider yourself a lonely person?	( ) completely lonely	( ) lonely	( ) a little lonely	( ) I do not feel lonely at all
19. To what extent do you consider yourself a lonely person even when you are at home with your family?	( ) completely lonely	( ) lonely	( ) a little lonely	( ) I do not feel lonely at all
20. To what extent do you consider yourself a lonely person even when you are at school?	( ) completely lonely	( ) lonely	( ) a little lonely	( ) I do not feel lonely at all
