# Supplementary material for: Social Isolation in Turkish Adolescents: Translation, Cross-Cultural Adaptation, and Validation of the Social Isolation Questionnaire
Source: Children (Basel). 2025 Aug 26;12(9):1122. doi: 10.3390/children12091122 (PMC12469003; doi:10.3390/children12091122)
Supplement: Supplementary file 1 [file children-12-01122-s001.zip › Supplementary_Turkish scale form.pdf]

Supplementary –Social Isolation Questionnaire (Turkish form)

1. Son 12 ay içinde kendinizi ne sıklıkla yalnız hissettiniz?	( ) Hiçbir zaman	( ) Nadiren	( ) Bazen	( ) Çoğu zaman	( ) Her zaman
2. Kaç yakın arkadaşın var? (Yakın arkadaşlar, ihtiyacın olduğunda seni dinleyebilecek veya sana yardım edebilecek kişilerdir.	( ) Yok	( ) 1	( ) 2	( ) 3	( ) 4 veya daha fazla
3. Sahip olduğunuz arkadaş sayısının ne kadar yeterli olduğunu düşünüyorsunuz ve ihtiyaç duyduğunuzda sizi dinleyecek veya yardım edecek kime güvenebilirsiniz?	( ) Tamamen yetersiz	( ) Yetersiz	( ) Yeterli	( ) Tamamen yeterli	
4. İhtiyacınız olduğunda sizi dinleyecek veya yardıma ihtiyacınız olduğunda size yardımcı olacak yakınlarınızın sayısını ne ölçüde yeterli görüyorsunuz?	( ) Tamamen yetersiz	( ) Yetersiz	( ) Yeterli	( ) Tamamen yeterli	
5. İnsanlarla olan ilişkilerinizi ve sosyal temaslarınızı ne kadar yüzeysel buluyorsunuz?	( ) Tamamen yüzeysel	( ) Yüzeysel	( ) Biraz yüzeysel	( ) Hiç yüzeysel değil	
6. Sorunlarınız ve duygularınız hakkında ebeveynlerinizle konuşma sıklığınızı ne ölçüde yeterli buluyorsunuz?	( ) Tamamen yetersiz	( ) Yetersiz	( ) Yeterli	( ) Tamamen yeterli	
7. Yakın arkadaşlarınızla sorunlarınız ve duygularınız hakkında konuşma sıklığını ne ölçüde yeterli buluyorsunuz?	( ) Tamamen yetersiz	( ) Yetersiz	( ) Yeterli	( ) Tamamen yeterli	
8. Öğretmenleriniz ve okul personeliyle sorunlarınız ve duygularınız hakkında konuşma sıklığınızı ne ölçüde yeterli buluyorsunuz?	( ) Tamamen yetersiz	( ) Yetersiz	( ) Yeterli	( ) Tamamen yeterli	
9. Yakınlarınızın önemli bir karar almaları gerektiğinde veya sorun yaşadıklarında sizinle görüşmek için size başvurma veya sizi arama sıklığını ne ölçüde tatmin edici buluyorsunuz?	( ) Tamamen yetersiz	( ) Yetersiz	( ) Yeterli	( ) Tamamen yeterli	
10. Arkadaşlarınızın, önemli bir karar almaları gerektiğinde veya sorunları olduğunda sizinle konuşmak için size danışma sıklığını ne kadar tatmin edici buluyorsunuz?	( ) Tamamen yetersiz	( ) Yetersiz	( ) Yeterli	( ) Tamamen yeterli	
11. Çevrenizdeki insanların fikirlerinizi paylaşmadığını veya ilgisiz olduğunu ne ölçüde düşünüyorsunuz?	( ) Hiç kimse ilgilenmiyor veya fikirlerimi paylaşmıyor	( ) Birkaç kişi ilgileniyor veya fikirlerimi paylaşıyor	( ) Çoğu insan ilgileniyor veya fikirlerimi paylaşıyor	( ) Herkes ilgileniyor veya fikirlerimi paylaşıyor	
12. En yakın arkadaşlarınızla olan ilişkilerinizin kalitesini ne kadar tatmin edici buluyorsunuz?	( ) Tamamen tatmin edici değil	( ) Tatmin edici değil	( ) Tatmin edici	( ) Tamamen tatmin edici	
13. Aile üyelerinizle olan ilişkilerinizin kalitesini ne ölçüde tatmin edici buluyorsunuz?	( ) Tamamen tatmin edici değil	( ) Tatmin edici değil	( ) Tatmin edici	( ) Tamamen tatmin edici	
14. Öğretmenleriniz ve diğer okul çalışanlarıyla kurduğunuz ilişkilerin kalitesini ne ölçüde tatmin edici buluyorsunuz?	( ) Tamamen tatmin edici değil	( ) Tatmin edici değil	( ) Tatmin edici	( ) Tamamen tatmin edici	
15. Tek başınıza bir şeyler yapmaktan veya başkaları tarafından dışlanmaktan, izole edilmekten veya engellenmekten ne kadar mutsuz oluyorsunuz?	( ) Tamamen mutsuz	( ) Mutsuz	( ) Biraz mutsuz	( ) Hiç mutsuz hissetmiyorum	
16. Kendinizi ne ölçüde başkaları tarafından dışlanmış, izole edilmiş veya engellenmiş olarak görüyorsunuz?	( ) Tamamen dışlanmış, engellenmiş hissediyorum	( ) Çok dışlanmış, engellenmiş hissediyorum	( ) Biraz dışlanmış, engellenmiş hissediyorum	( ) Dışlanmış, engellenmiş hissetmiyorum	

17. Çevrenizdeki insanlara yaklaşma ve iletişim kurma beceriniz sizi ne ölçüde rahatsız ediyor?	( ) Beni çok rahatsız ediyor	( ) Beni rahatsız ediyor	( ) Beni biraz rahatsız ediyor	( ) Hiç rahatsız etmiyor
18. Genel olarak kendinizi ne ölçüde yalnız bir insan olarak görüyorsunuz?	( ) Tamamen yalnız	( ) Yalnız	( ) Biraz yalnız	( ) Hiç yalnız hissetmiyorum
19. Ailenizle birlikte olduğunuzda bile kendinizi ne ölçüde yalnız bir insan olarak görüyorsunuz?	( ) Tamamen yalnız	( ) Yalnız	( ) Biraz yalnız	( ) Hiç yalnız hissetmiyorum
20. Okuldayken bile kendinizi ne kadar yalnız bir insan olarak görüyorsunuz?	( ) Tamamen yalnız	( ) Yalnız	( ) Biraz yalnız	( ) Hiç yalnız hissetmiyorum

Scoring the QIS questionnaire

The following steps were used to calculate the overall score of the questionnaire:

Step 1. Question scores: The alternatives of each question were assigned values from 0 to 3: 0 without characteristics of social isolation and 3 with strong characteristics of social isolation;

Step 2. Scores for each dimension: The values of the questions included in each dimension were added up;

Step 3. QIS score: The value of each dimension (Step 2) was multiplied by its weight within the QIS and then summed, resulting in the following formula:

$$QIS = (Q1+Q15+Q16+Q17+Q18+Q19+Q20)*3.0 + (Q2+Q3+Q7+Q10+Q11+Q12)*2.0 + (Q4+Q6+Q9+Q13)*1.5$$

The QIS score can assume values from 0 to 131, with 0 characterizing an adolescent with a minimum score of social isolation, and 131 an adolescent with a maximum score of social isolation.

Note: Reference: Dos Santos SJ, Soares FC, Gaoua N, Rangel Junior JF, Lima RA, de Barros MVG. Development and validation of a scale to measure social isolation in adolescents. J Res Adolesc. 2024;34(3):1069-77. https://doi.org/10.1111/jora.12952
